# Detecting blood clots with aptamers: A potentially lifesaving new tool in medicine

**DOI:** 10.1016/j.omtn.2023.02.018

**Published:** 2023-03-06

**Authors:** John J. Rossi

**Affiliations:** 1 Center for RNA Biology and Therapeutics, City of Hope, Monrovia, CA 91016, USA

Blood clots are a serious problem that can lead to death or serious disability following an ischemic stroke. Current methods detect reduced blood flow due to clots forming in an artery. The detection of clots before they block blood flow is important in the rapid treatment of patients undergoing surgical procedures or treatments for cancer. In this issue of *Molecular Therapy - Nucleic Acids*, Gray et al.^1^ describe an RNA aptamer targeting the clot-associated protein thrombin that has been adapted for detection of blood clots in real time.

Aptamers are polynucleic acids that can fold into three-dimensional structures that bind ligands with high affinity.^2^ The process by which aptamers can be created is called SELEX (systematic evolution of ligands by exponential enrichment).^2^ Since the first description of this technique was published, thousands of aptamers have been evolved that bind to their ligands with sub-nanomolar affinity.

Sullenger and colleagues have pioneered aptamer development for the treatment of cardiovascular diseases such as ischemic stroke.^3^ Moreover, their rationale for aptamers and blood clotting regulation was that the binding of aptamers to the target ligand could be rapidly reversed by treatment with antidotes consisting of single-stranded oligonucleotides, which bind to the aptamer via complementary base-pairing, thereby perturbing the aptamer structure sufficiently to reverse the binding.^3^

Gray et al.^1^ extended the anti-clotting aptamer concept. This study demonstrates that a non-therapeutic aptamer that selectively binds to thrombin can be used to visualize clot formation *in vivo*. Thrombin is only found associated with blood clots once it is cleaved from its precursor prothrombin.

Early detection of a clot can lead to rapid anti-clotting treatments and save lives as well as major tissue destruction caused by reduced blood flow. The thrombin recognition is indicative of an active clot. These investigators labeled the thrombin aptamer with a near-infrared dye, facilitating detection of the thrombin-/clot-bound aptamer. Moreover, unbound aptamers were readily eliminated from circulation with the use of an antidote antisense oligonucleotide, which rapidly binds to the aptamer and disrupts its structure.

The salient features of the aptamer are that it was evolved to selectively bind human thrombin, which is only present at the site of clot formation. Moreover, the human aptamer cross-reacted with murine thrombin, allowing *in vivo* visualization in mice induced to undergo thrombosis. They extended the testing of the thrombin clot detecting aptamer to porcine and non-human primate models.

In clinical applications, this aptamer can be used during surgical procedures, which often trigger blood clot formation, and in patients with cancer undergoing chemotherapy with clotting risks. The complete reversal of the aptamer in circulation and at the clot itself with the antidote antisense oligonucleotide is an important aspect of this process. Real-time detection of clot formation can lead to rapid treatment with agents such as tissue plasminogen activator TPA, thereby saving lives and reducing the disabilities associated with ischemic strokes. Overall, this remarkable tool for detecting thrombosis once again demonstrates the power of nucleic acids as both diagnostic and therapeutic agents.
